# GOmotif: A web server for investigating the biological role of protein sequence motifs

**DOI:** 10.1186/1471-2105-12-379

**Published:** 2011-09-26

**Authors:** Franklin Bristow, Runtao He, Gary Van Domselaar

**Affiliations:** 1 National Microbiology Laboratory, Public Health Agency of Canada, Winnipeg, MB, R3E 3R2, Canada; 2 Department of Medical Microbiology, School of Medicine, University of Manitoba, Winnipeg, MB, R3T 2N2, Canada; 3 Department of Computer Science, University of Manitoba, R3T 2N2, Winnipeg, MB, Canada

## Abstract

**Background:**

Many proteins contain conserved sequence patterns (motifs) that contribute to their functionality. The process of experimentally identifying and validating novel protein motifs can be difficult, expensive, and time consuming. A means for helping to identify in advance the possible function of a novel motif is important to test hypotheses concerning the biological relevance of these motifs, thus reducing experimental trial-and-error.

**Results:**

GOmotif accepts PROSITE and regular expression formatted motifs as input and searches a Gene Ontology annotated protein database using motif search tools. The search returns the set of proteins containing matching motifs and their associated Gene Ontology terms. These results are presented as: 1) a hierarchical, navigable tree separated into the three Gene Ontology biological domains - biological process, cellular component, and molecular function; 2) corresponding pie charts indicating raw and statistically adjusted distributions of the results, and 3) an interactive graphical network view depicting the location of the results in the Gene Ontology.

**Conclusions:**

GOmotif is a web-based tool designed to assist researchers in investigating the biological role of novel protein motifs. GOmotif can be freely accessed at http://www.gomotif.ca

## Background

Protein sequence motifs are conserved patterns of amino acids that have some sort of biological significance [1]. One example of a well-known protein sequence motif is the zinc finger motif [2]. Each zinc finger is composed of about 30 residues described by the consensus pattern Cys-X2-4-Cys-X3-Phe-X5-Leu-X2-His-X3-His (where X is any amino acid). The zinc finger motif folds into a ββα structure through hydrophobic interactions and coordination of a zinc ion by two conserved cysteine and histidine residues. The zinc finger typically recognizes a 3 bp stretch of DNA sequence. Its discovery in transcription factor TFIIIA from Xenopus laevis was facilitated in part by its repeated occurrence in the protein [2].

There are many other motifs that are involved in important biological processes including protein-protein interactions, protein-nucleic acid interactions, post-translational modification, protein trafficking, signal transduction, and others. The PROSITE database contains a large curated and documented collection of these motifs [3]. The patterns describing these motifs often are not as conserved or as easily discovered as the zinc finger motif. For example, the LXXLL/LLXXL motif has been shown to play an important role in protein-protein interactions in nuclear receptor co-activators [4]. The SXXXXS and AXXXXA motifs have also been found to be involved in protein-protein interactions [5].

Currently, the commonly used methods to characterize novel motifs are through experimental procedures, such as yeast two-hybrid systems, mammalian two-hybrid systems, crystallography, and mass spectrometry [6-9]. These methods, though powerful, can be expensive, time consuming, can have poor reproducibility, and are subject to trial-and-error. A means for helping to identify in advance the possible function of a novel motif is desirable to generate hypotheses and to support existing hypotheses concerning the biological relevance of these motifs, reducing experimental trial-and-error.

Novel protein sequence motif candidates are typically identified by grouping several sequences together using similarity search tools like BLAST [10], CLUSTALW [11] or using motif discovery tools like the MEME Suite [12], DILIMOT [13], SLiMFinder [14], or FIRE-Pro [15]. The conserved regions within a group of sequences can then be described as a pattern of amino acids and gaps at certain locations where multiple residues may be matched at any position and there may be variable sized gaps [1]. Once identified, the next step is to characterize the biological relevance of a motif.

Ultimately, ascertaining the biological relevance of a novel motif requires experimental validation which, as discussed, is often prohibitive, both in terms of time and cost. Therefore, searching a larger database of protein sequences prior to validation to determine if there is a significant association with a specific biological role is advantageous. Resources exist that allow a researcher to search a database of protein sequences for sequence motifs [16], but inferring biological relevance from the results can be difficult if the functional descriptions are inconsistently annotated in terms of their descriptive text and functional specificity - often the case for biological sequence databases. In order to find a consistent association of a sequence pattern with a biological role that is computationally straightforward, an organized, controlled vocabulary is required. Fortunately, this resource exists in the form of the Gene Ontology [17].

Founded by FlyBase, the Saccharomyces Genome Database, and the Mouse Genome Database in 1998, the Gene Ontology Consortium provides a standardized and controlled vocabulary for describing genes and gene products in any organism. The controlled vocabulary, commonly known as the Gene Ontology (GO), is represented as a directed acyclic graph (DAG) [17]. GO provides hierarchies for three biological domains: *cellular component*, *biological process*, and *molecular function*. Beneath these biological domains are increasingly specific, hierarchically arranged descriptions, the most descriptive terms being the leaf nodes of the DAG. The controlled vocabulary of the GO provides a consistent reference for describing the function of protein sequence motifs and the hierarchical organization of the GO lends to intuitive visualization of the relationship of these descriptions in the form of a graph.

Several tools already exist that are capable of putatively assigning GO terms to protein sequences [18-21]. As an example, Blast2GO assigns GO terms to individual sequences on the basis of sequence similarity. Input sequences are aligned against a database having existing GO annotations and are then transitively assigned a function based upon their similarity to the sequences in the database [19]. Blast2GO also provides useful tools for visualizing the location of the query sequences in the Gene Ontology DAG. Blast2GO however is not ideal for finding protein sequence motifs as the BLAST algorithm is designed for quickly finding the overall similarity between a set of sequences rather than a defined pattern contained within them. Methods have been developed to predict the biological significance of protein sequence motifs [22] but have not been implemented in a software tool that allows researchers to predict the biological significance of a novel motif.

We present here the GOmotif website for investigating the biological significance of novel protein sequence motifs. GOmotif provides a highly interactive user interface that researchers can use to investigate the distribution of GO terms associated with proteins in the SwissProt database [23] that are returned from a motif search. The search results are presented in several forms, including: 1) a hierarchical tree structure; 2) pie charts that are created by GOmotif indicating the number of hits to each sub-graph within the GO; and 3) an interactive graph viewer showing the tree structure of the GO terms associated with the search results. GOmotif has been designed to be an easy to use tool to complement traditional methods of assigning function to motifs. As a web application, the only requirement to use GOmotif is an internet connection and a modern web-browser with support for Java applets.

## Implementation

GOmotif is a web service written in Java using several different technologies. The GUI for GOmotif was written in Java using the Echo presentation toolkit along with GraphViz [24] and ZGRViewer [25] for graph visualizations. GOmotif also uses BioJava [26] to parse sequence databases and ScanProsite [16] to scan databases using PROSITE-formatted motifs.

## Results and Discussion

### Input, Analysis and Presentation of Results

GOmotif accepts as input one or more PROSITE or regular expression formatted protein sequence motifs. Additional options allow a researcher to restrict the location of the motif with reference to the N or C terminus, to include or exclude overlapping motifs, and to specify a taxonomic subset within which to restrict the search. All input data and options are validated before processing.

GOmotif searches the submitted sequence pattern(s) against the SwissProt database of expertly curated protein sequences using ScanProsite [16]. GOmotif then extracts the associated GO terms from the SwissProt records and analyzes them for their statistical overrepresentation in the result set using a method similar to the one used in BiNGO [18] or GO::TermFinder [27].

**Figure 1:**
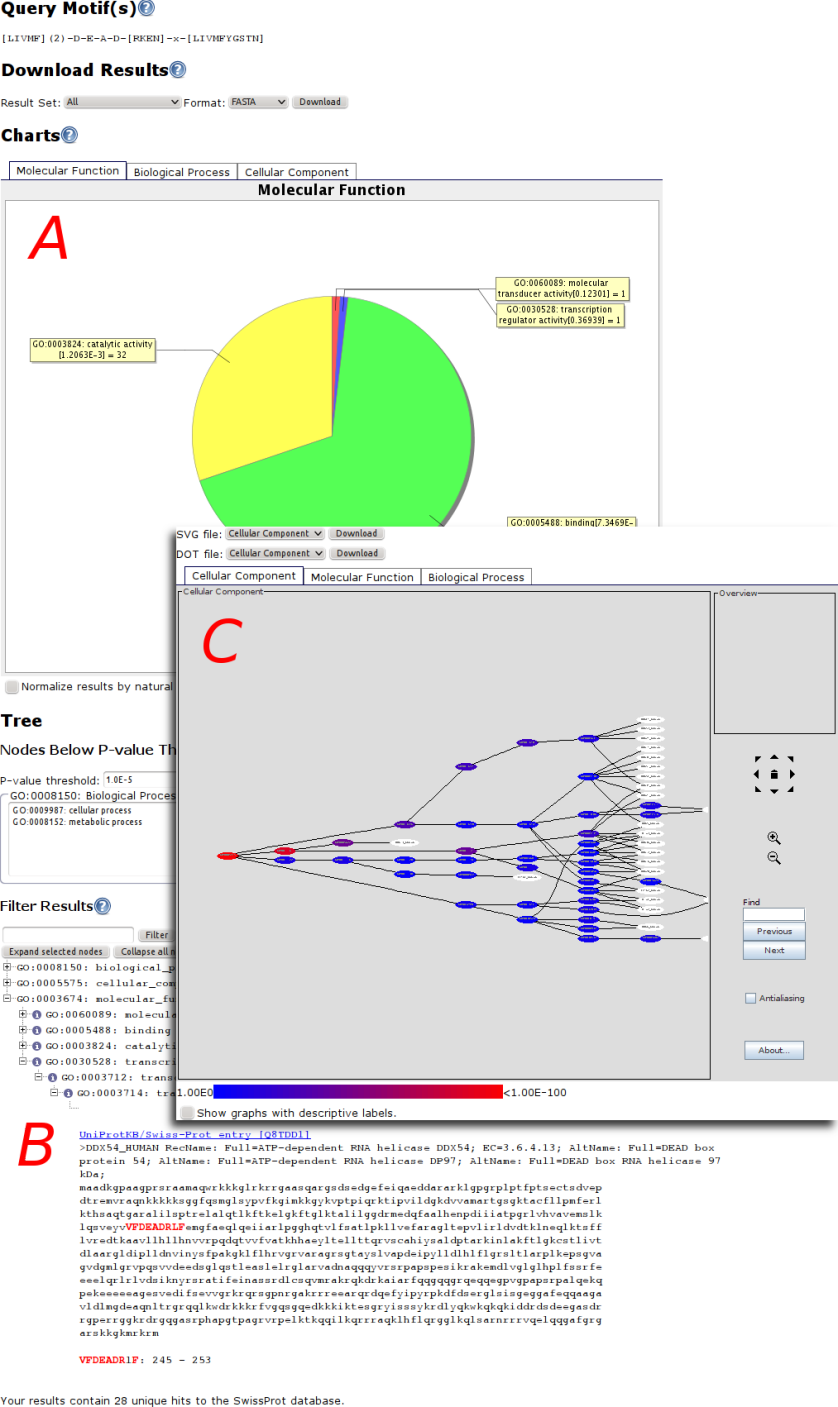
**The GOmotif report**. The three different types of results shown by GOmotif can be seen in Figure 1, including A) the pie chart view, B) the GO tree view, and C) the GO graph view.

Once a submission has completed processing, GOmotif presents the results in three different ways (Figure 1): 1) a graphical pie chart view indicating the distribution of matching sequences for each of the major components of the Gene Ontology, 2) a hierarchical, interactive tree view showing the FASTA formatted results and their location in the Gene Ontology hierarchy and, 3) an interactive, zoomable graph view showing the actual location of Gene Ontology terms and SwissProt hits in the Gene Ontology graph. If GOmotif finds GO terms that are significantly associated with the motif search they will be presented as well.

The simplest view that GOmotif offers is a graphical pie chart indicating the number of GO terms associated with that query motif and their distribution within the high-level GO categories (Figure 1A). There are three pie charts that are generated by GOmotif, each of which corresponds to one of the three main biological domains of the Gene Ontology: *biological process*, *molecular function*, and *cellular component*. These charts are displayed to the investigator as an image in a set of three tabs, such that one pie chart is visible at any given time. Each of the pie charts displays a broad level subset of each of the domains. Biological process and molecular function are displayed using 'GO Slim' sets (i.e., a single level, broad subset of the terms contained in the entire GO); cellular component is displayed using a custom subset of terms that are one level below the cellular component GO term. By default the pie charts report the raw distribution of matching sequences from the results of the PROSITE. Investigators also have the option to "normalize" these charts. In other words, the charts can be redrawn in a way that the abundances reported for each of the terms in the chart are corrected for the distribution of GO terms in the SwissProt database (or taxonomic subset). This method of normalization attempts to correct for the skew that the raw number of entries and terms in the database might introduce into the results.

In addition to the distribution scores, the pie charts also show a statistical p-value that indicates the overrepresentation of a given term similar to the method used in BiNGO [18] and GO::TermFinder [27]. Larger p-values indicate that a term is not significantly overrepresented whereas smaller p-values indicate that a term is more highly overrepresented in a result set. The p-value is calculated using a hypergeometric distribution described by Boyle et al. [27], where the population size is the number of sequences in the SwissProt database, the number of successes is the total number of sequences in the SwissProt database annotated with a specific GO term, and the sample size is the number of SwissProt sequences returned by ScanProsite.

On this same page investigators can use the tree view (Fig 1B) to navigate the structure of the Gene Ontology graph. While the Gene Ontology itself is technically a directed acyclic graph, the graph can be displayed intuitively in a tree-like fashion. In this view, researchers are able to navigate through the tree structure to find exactly which sequences from the motif search were reported and where those hits are located. GOmotif makes use of the BioJava framework [26] for accessing and displaying sequence data. Each database hit that is returned by the motif pattern search can have more than one Gene Ontology annotation, thus each result can be located in several locations throughout the tree under any of the three GO domains.

The tree view can be filtered to remove any extraneous or undesired branches or nodes from the search results. The investigator can perform a text search to filter results in the database or entries in the tree view can be manually selected for removal. GOmotif dynamically updates the views to reflect the filtered search results. GOmotif also provides functionality for the user to expand an entire sub-tree of the results displayed, allowing for direct inspection of the protein sequences in the context of the GO tree.

The actual protein sequences are displayed in FASTA format within the tree view. The matching motifs are highlighted in red and are presented in upper-case so that they are plainly visible to the investigator. A link to the SwissProt/UniProtKB entry for each sequence is provided just above the sequence. GOmotif provides an option to download the set of filtered protein sequences returned from the motif search in FASTA and SwissProt formats.

In addition to the pie chart and tree view, GOmotif renders graphs of a reasonable size with GraphViz [24] and displays the graphs in ZGRViewer [18], a zoomable, interactive graphical interface. Researchers can view the Gene Ontology graphs constructed from the GO terms associated with the protein sequences from the motif search. Each GO term displayed in this interface is assigned a color based upon the p-value calculated to measure its statistical overrepresentation in the SwissProt database. A node that is red in color indicates that the node is significantly overrepresented; a blue node indicates that the GO term is not significantly overrepresented. Each node in the graph links to either the AmiGO entry for GO terms, or to the SwissProt/UniProtKB entry for protein sequences. The graph view provides the user with the option to download the graph in two formats: a DOT-formatted file suitable for use with GraphViz compatible applications (regardless of the result size), or an SVG-formatted file suitable for presentation and editing in 3^rd ^party applications.

## Validation

### Assessing GO Term Selection Accuracy

GOmotif reports all instances of statistically overrepresented GO terms by identifying GO terms in the result set below an adjustable, default threshold p-value of 10^-6^. These GO terms form a set of putative biological roles that can then be experimentally validated.

Given that the PROSITE database of protein motifs contains experimentally validated true positive and false positive motif matches to the protein records contained in the SwissProt database, we decided to use this information to assess the ability of our GO term selection scheme to correctly identify the biological significance of a motif search. Based on the assumption that a subset of GO terms for each protein record in SwissProt that is a true positive for a motif match, we transitively applied these true positive and false positive designations to the GO terms associated with the SwissProt records identified by PROSITE. GO terms in common with both the true positive and false positive records were considered unrelated and discarded from the analysis. We then processed each pattern entry in the PROSITE database using GOmotif and compared the predicted GO terms to the set of true positive and false positive GO terms assigned to the pattern. We define the number of true positive predictions as the number of predicted GO terms that match one of the true positive GO terms associated with the PROSITE pattern. We define a false positive as a predicted GO term that either matches a false positive GO term associated with the PROSITE pattern or a GO term that is not associated with the set of false positive or true positive GO terms for that pattern. We define the number of true negative predicted GO terms to be the number of false positive GO terms associated with the PROSITE pattern that were not selected by the prediction scheme. Finally, we define the number of false negatives as the number of true positive GO terms associated with the PROSITE pattern that were not selected by the prediction scheme. We then computed specificity and sensitivity for all pattern entries in the PROSITE database that listed both true and false positive SwissProt records and calculated the average sensitivity and specificity for each of the three main GO domains (a high-level report can be found in additional file 1, and detailed node selection reports can be found in additional files 2, 3 and 4).

**Figure 2:**
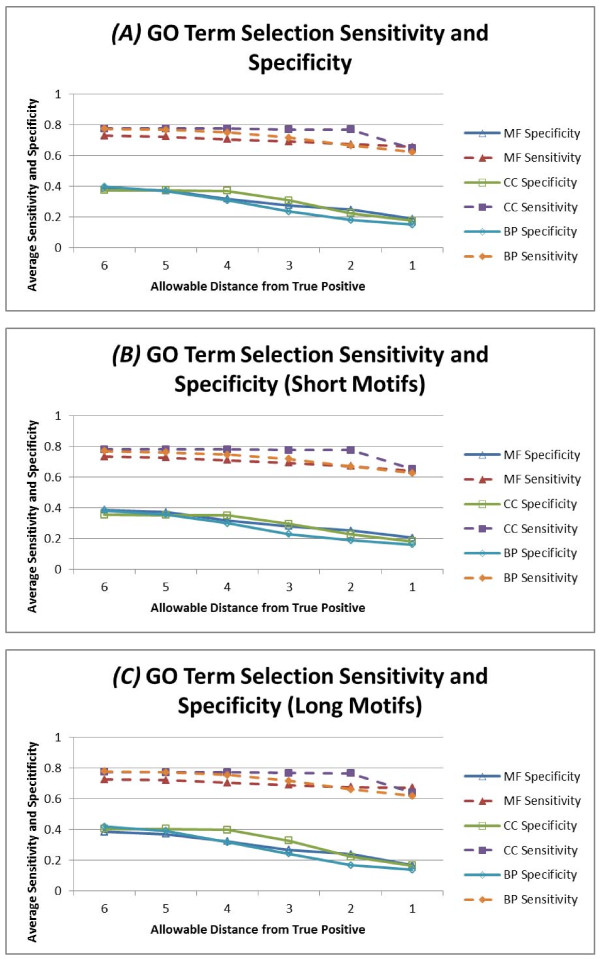
**Validation of GO term selection by sensitivity and specificity**. We show in (a) that the sensitivity and specificity of our GO term selection mechanism declines as we enforce a stricter definition of a true positive GO term selection. We show the sensitivity and specificity for each of the three biological domains: *molecular function (MF)*, *biological process (BP) *and *cellular component (CC)*. In (b) and (c) we show the changes in sensitivity and specificity when searching short and long motifs respectively. We attribute the increase in sensitivity and decrease in specificity for short motifs to the naturally larger number of records in a sequence database matched by a shorter motif, leading to fewer false negatives and more false positives. Values for these charts can be found in additional file 1, and detailed node selection reports used to generate this chart can be found in additional files 2, 3 and 4.

We measured the sensitivity and sensitivity of the selection algorithm executing searches for each PROSITE pattern entry. We calculated sensitivity and specificity for a variety of allowable distances from the "correct" GO term between 1 (i.e., we selected the correct GO term precisely, or a very strict definition of true positive) and 6 (i.e., a selection of any GO term up to 6 nodes away from the correct GO term, or a very loose definition of true positive). Furthermore, we calculated average sensitivity and specificity separately for short motifs (patterns with length less than 15), and long motifs (the remaining patterns in the PROSITE database). Figure 2A shows the average sensitivity and specificity values calculated. The specificity of our selection mechanism is consistently low, indicating that we are often making false positive selections of GO terms; however the sensitivity of our selection mechanism is consistently high indicating that we accurately choose a significant number of true positive GO terms associated with a PROSITE pattern. Figures 2B and 2C show the average sensitivity and specificity calculated for short and long motifs, respectively. The average sensitivity is noticeably higher for shorter motifs. Since shorter motifs will naturally match more records in the sequence database by random chance, the number of false negatives decreases. The average specificity for shorter motifs is lower for the same reasons - the increased number of matches in the sequence database increases the total number of false positive GO terms selected for shorter motifs. Because the false positive rate is substantial, it is important for researchers to include their prior knowledge of the possible role(s) for a submitted motif when assessing the results of a GOmotif search.

Our assessment of the ability of overrepresented GO terms to predict the biological significance of a protein sequence motif yields reasonable predictive accuracies; however, it must be noted that this does not mean that one can assume that the biological significance associated with an overrepresented GO term is caused by the motif. For this reason GOmotif should be used only as an investigational tool to identify possible biological roles for a novel motif. Ultimately the biological significance of any novel protein sequence motif must be experimentally verified.

### Improving Results by Taxonomic Subset

The biological role of a protein sequence motif is a function not only of the motif but also of the biological environment in which it exists. As organisms evolve the biological role of a sequence motif may evolve as well. To accommodate this phenomenon GOmotif provides facilities for restricting the motif search to specific taxonomies.

In order to demonstrate the ability to improve the results by restricting the search to a specific taxonomic subset we selected a protein sequence motif from the PROSITE database that had a large number of false positive hits when searching through the entire SwissProt database. Selecting a PROSITE entry with many false positives allowed us to ensure that specifying taxonomy could successfully help in determining the function of a motif if the taxonomy it belonged to was known. The specific PROSITE entry that was chosen for this purpose was the Sigma-54 interaction domain ATP-binding region A signature (PROSITE id PS00675). This entry was selected because of its specificity for prokaryotes and its high number of false positive hits (this motif has a precision of only about 31% when searched against all taxonomies).

**Figure 3:**
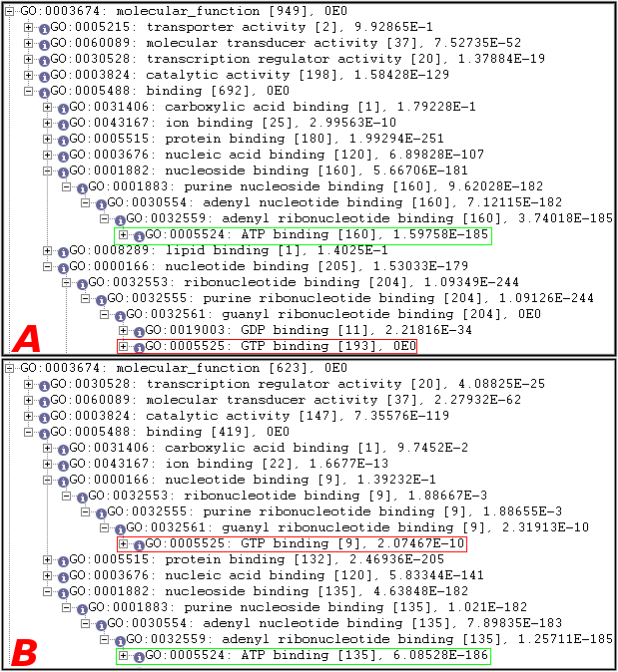
**A portion of the results from a search of a motif describing the "Sigma-54 interaction domain ATP-binding region A signature" (PS00675) motif pattern against (a) all entries in the SwissProt database and (b) the bacterial taxonomy**. PS00675 is a motif pattern describing ATP binding signatures of bacteria and has a large number of false positive eukaryotic hits. In (a) we show that by searching the entire SwissProt database no clear distinction can be made between the significance of the ATP binding GO term and the GTP binding GO term. In (b) we show that restricting the taxonomic subset of the motif search to the bacterial taxonomy we reduce the number of false positive eukaryotic hits and show that the ATP binding GO term clearly has a greater significance than the GTP binding GO term.

Figure 3A shows a portion of the results returned after searching for this motif against all entries in the SwissProt database. This motif appears to have a significant number of results that with either ATP binding or GTP binding. Upon further inspection, the vast majority of the results that are being returned for GTP binding are eukaryotes that are false positive matches for this particular motif.

Figure 3B shows a portion of the results returned after searching for this motif and restricting the taxonomy to prokaryotes. The set of results provided with this query show that ATP binding is a far more significant result than GTP binding. Restricting the taxonomy to prokaryotes increased the accuracy from about 31% to about 80%.

## Conclusion

GOmotif is a tool designed to assist in investigating the possible the biological role(s) of protein sequence patterns. Researchers can submit one or more motifs, search the SwissProt database of protein sequences (or a taxonomic subset of the database) for protein sequences containing matching motifs, and use a number of helpful visualizations of the GO terms associated with the search results to investigate the relationship between the submitted motifs and *molecular function*, *biological process*, and *cellular component *of protein sequences with matching motifs using a number of helpful visualizations of the GO terms associated with the search results. GOmotif is not a tool for predicting the biological significance of a novel sequence motif, however, GOmotif can be useful to test hypotheses about the biological relevance of sequence motifs and thus guide biologists in the assigning biological roles to the sequence patterns that they investigate.

## Availability and Requirements

**Project name: **GOmotif

**Project home page: **http://www.gomotif.ca

**Operating system(s): **Platform Independent

**Programming language: **Java, Perl

**Other Requirements: **Java 5 or higher, Tomcat 6.0 or higher (Running local server), a recent web browser with support for Java Applets (Running searches remotely).

**License: **GNU GPL

**Any restrictions to use by non-academics: **None

## Authors' contributions

FB designed and implemented the software used in this project. RH participated in the design of the application and helped draft the manuscript. GVD conceived of the study, participated in its design and coordination and helped draft the manuscript. All authors read and approved the final manuscript.

## Supplementary Material

Additional File 1 **Overall Average Sensitivity and Specificity**. This spreadsheet reports the overall specificity and sensitivity calculated for each of the three biological domains (*molecular function*, *biological process *and *cellular component*). This spreadsheet provides a high-level overview of the data contained in the remaining additional files 2, 3 and 4. Click here for file

Additional File 2 **Node selection report for *Biological Process***. A report of the fine-grained details about how sensitivity and specificity were calculated for *Biological Process*. Column A (Allowed Distance) reports the allowable distance from the correct GO term that can be reported as a true positive. Column B (PROSITE ID) reports the PROSITE database identifier that provided these results. Column C reports the PROSITE pattern record for this PROSITE ID, and column D reports the length of the PROSITE pattern. Column E (All Selected) reports the total number of GO terms that were selected by our selection algorithm. Column F-I (True Positives, True Negatives, False Positives, False Negatives) reports the total number of GO terms selected that match the criteria for true positive, false positive, true negative or false negative. Columns J and K (Specificity and Sensitivity) report the calculated specificity and sensitivity for the data in the row. Click here for file

Additional File 3 **Node selection report for *Molecular Function***. A report of the fine-grained details about how sensitivity and specificity were calculated for *Molecular Function*. Column A (Allowed Distance) reports the allowable distance from the correct GO term that can be reported as a true positive. Column B (PROSITE ID) reports the PROSITE database identifier that provided these results. Column C reports the PROSITE pattern record for this PROSITE ID, and column D reports the length of the PROSITE pattern. Column E (All Selected) reports the total number of GO terms that were selected by our selection algorithm. Column F-I (True Positives, True Negatives, False Positives, False Negatives) reports the total number of GO terms selected that match the criteria for true positive, false positive, true negative or false negative. Columns J and K (Specificity and Sensitivity) report the calculated specificity and sensitivity for the data in the row. Click here for file

Additional File 4 **Node selection report for *Cellular Component***. A report of the fine-grained details about how sensitivity and specificity were calculated for *Cellular Component*. Column A (Allowed Distance) reports the allowable distance from the correct GO term that can be reported as a true positive. Column B (PROSITE ID) reports the PROSITE database identifier that provided these results. Column C reports the PROSITE pattern record for this PROSITE ID, and column D reports the length of the PROSITE pattern. Column E (All Selected) reports the total number of GO terms that were selected by our selection algorithm. Column F-I (True Positives, True Negatives, False Positives, False Negatives) reports the total number of GO terms selected that match the criteria for true positive, false positive, true negative or false negative. Columns J and K (Specificity and Sensitivity) report the calculated specificity and sensitivity for the data in the row. Click here for file
